# Speckle Tracking Stress Echocardiography Uncovers Early Subclinical Cardiac Involvement in Pediatric Patients with Inflammatory Bowel Diseases

**DOI:** 10.1038/s41598-017-03255-1

**Published:** 2017-06-07

**Authors:** Kai O. Hensel, Francisca E. Abellan Schneyder, Lucia Wilke, Andreas Heusch, Stefan Wirth, Andreas C. Jenke

**Affiliations:** 1 HELIOS University Medical Center Wuppertal, Children’s Hospital, Department of Pediatric Gastroenterology, Center for Clinical & Translational Research (CCTR), Faculty of Health, Center for Biomedical Education & Research (ZBAF), Witten/Herdecke University, Faculty of Health, Witten, Germany; 2 HELIOS University Medical Center Wuppertal, Children’s Hospital, Department of Pediatric Cardiology, Center for Clinical & Translational Research (CCTR), Faculty of Health, Center for Biomedical Education & Research (ZBAF), Witten/Herdecke University, Faculty of Health, Witten, Germany; 3 EKO Children’s Hospital, Department of Pediatric Gastroenterology, Oberhausen, Witten/Herdecke University, Faculty of Health, Witten, Germany

## Abstract

Inflammatory bowel disease (IBD) is an established risk factor for cardiovascular disease (CVD). However, whether cardiac consequences present early in IBD is unknown. This is the first study in children aiming to unmask altered myocardial mechanics in IBD. We enrolled 50 consecutive normotensive children with Crohn’s disease (CD) (n = 28) or ulcerative colitis (UC) (n = 22). The study groups consisted of 18 patients with active inflammatory disease (mean age 14.6 ± 2.5 years) and 32 children with IBD in remission (14.3 ± 2.3 years). 60 age- and gender-matched children served as healthy controls. Speckle tracking stress echocardiography (STE) was used to assess left ventricular (LV) myocardial strain and strain rate. Circumferential strain rate was significantly decreased in children with active IBD (−1.55 ± 0.26 s^−1^) and IBD in remission (−1.49 ± 0.26 s^−1^) versus healthy controls (1.8 ± 0.4 s^−1^) both at rest (p < 0.001) and during exercise (p = 0.021). Moreover, longitudinal strain rate, circumferential strain and E/E′ ratio were significantly impaired in IBD. Pediatric patients with IBD feature subclinical signs of LV systolic and diastolic myocardial impairment early in the course of CD and UC. This may not be reversible even when IBD is clinically controlled. Patients with IBD should be regularly screened for signs of CVD.

## Introduction

Inflammatory bowel diseases – essentially comprised of Crohn’s disease (CD)^1^ and ulcerative colitis (UC)^2^ – are a major health problem worldwide with ever-increasing incidence^3, 4^. While CD and UC are characterized by specific, clinically different characteristics, both diseases have a chronically-remitting inflammatory process of the gastrointestinal tract as well as potential extra-intestinal manifestations in common^5, 6^. IBD is an established risk factor for cardiovascular disease (CVD)^7^. Patients with IBD were shown to have a twofold to threefold increased risk for the development of venous thromboembolism^8^, specifically during acute disease flares when a disturbed homeostasis of anti- and procoagulants results in a hypofibrinolytic state^9^. Other known concomitant cardiovascular (CV) manifestations of CD and UC include Takayasu’s arteritis^10, 11^, pericarditis and myocarditis^12, 13^. The predominant underlying processes of CV pathology in IBD are currently thought to be the chronic exposure to the persistent/remittant inflammation and an altered lipid metabolism on the one hand as well as undesired adverse effects of long-term administered antiinflammatory drugs such as corticosteroids, TNF-α inhibitors, etc. on the other hand^14^. Furthermore, chronic inflammation has been demonstrated to be accompanied by pathological collagen disposition in affected organs^15, 16^. Whether this pathologically altered collagen disposition process also affects other organs, e.g. the heart in IBD, yet remains to be illuminated. Interestingly, studies in both adult and pediatric patients have demonstrated an association of IBD and early signs of subclinical atherosclerosis^17, 18^. Moreover, Doppler-based ultrasound studies have been utilized to reveal both increased arterial stiffness and carotid intima-media-thickness (IMT) in adult patients with CU^19^. Conventional echocardiographic studies focusing on left ventricular (LV) function, however, have yet failed to convincingly detect overt cardiac involvement in patients with IBD without other confounding CV risk factors.

Speckle tracking echocardiography (STE) is an advanced echocardiographic methodology for the quantification of myocardial function^20^. STE has been successfully used to identify early subclinical cardiac involvement in a variety of asymptomatic populations with chronic diseases and unremarkable conventional echocardiography. Representative examples include adult patients with arterial hypertension^21^ and children with uncomplicated type 1 diabetes mellitus^22^. Furthermore, STE is sensitive enough to detect discretely altered myocardial contractility due to transient changes in serum glucose levels^23^. Studies in adult patients have utilized STE to demonstrate subclinical cardiac impairment in both UC^24, 25^ and CD^25, 26^. However, whether myocardial function is already pathologically changed in pediatric patients with IBD is still unknown. This is the first study in children aiming to utilize STE in CD, UC and healthy controls to uncover subclinical myocardial impairment in an early state of IBD.

## Methods

### Study population

110 children aged 6 to 17 years were enrolled for this study. The study group consisted of 18 consecutive patients with active IBD (mean age 14.6 ± 2.5 years; 50% female) and 32 patients with IBD in remission (mean age 14.3 ± 2.3 years; 46.9% female) who are being followed up at the Pediatric Gastroenterology Clinic at Helios University Medical Centre Wuppertal, Germany. 60 healthy age- and sex-matched volunteers (mean age 14.0 ± 2.5 years; 61.6% female) served as the control group. 22 patients had been diagnosed with CD (mean age 15.0 ± 2.4 years; mean disease duration 2.9 ± 3.0 years; 46.4% female) and 28 had UC (mean age 14.0 ± 2.3 years; mean disease duration 2.8 ± 3.4 years; 45.5% female). Inclusion criteria for the study group were the definite diagnosis of CD or UC, which had been based on standard clinical, radiological, endoscopic and histological criteria findings in accordance with the revised Porto criteria and Montreal classification^27, 28^. Study group patients were stratified into sub-groups of active disease and IBD in remission by two experienced pediatric gastroenterologists according to disease activity indices, endoscopy results, degree of mucosal healing, clinical course and individual well-being. Primary exclusion criteria were other past or present health conditions likely affecting the cardiovascular system such as LV dysfunction, acquired valvular disease, congenital heart disease, kidney disease, developmental delay, body mass index >30 kg/m^2^, pathologic ECG-changes at rest or during exercise as well as technical limitations such as poor echocardiographic image quality, submaximal effort during exercise testing or short leg length. In addition, all patients with unclear underlying disease were excluded from the study. Several primarily enrolled patients were excluded from the study during the echocardiographic examination due to valvular disease (n = 1), poor echocardiographic image quality (n = 12) or inadequate cycling effort (n = 2).

Healthy controls had a completely negative medical history both regarding the cardiovascular and gastrointestinal as well as any other organ system. Initially, a thorough history and physical examination as well as both resting and exercise standard echocardiography and ECG were obtained in all study subjects. Every participant as well as their legal guardian signed a written informed consent prior to inclusion in the study. The study sample size was achieved by enrolling all patients from the hospital’s Pediatric Gastroenterology-Inflammatory Bowel Diseases Section who agreed to participate. A priori, a study design was established dividing the study population into subgroups of IBD patients with acute inflammation and clinical remission. The study was carried out in accordance with the declaration of Helsinki’s ethical principles for medical research involving human subjects and approved by the Witten/Herdecke University ethics committee (*clinical trial number: 103/2014*).

Laboratory findings were obtained at the day of the study visit or acquired from the patient record when they had been obtained within two weeks prior to the echocardiographic investigation to minimize percutaneous punctures in this pediatric study population.

### Evaluation of disease severity

In 25 patients, a standard Mayo score was utilized^29^. The partial Mayo score was used for all other UC patients, in which diagnostic endoscopy had taken place more than four weeks prior to the echocardiographic study. It consists of three of the four original Mayo score parameters (pediatric physician’s overall assignment, stool frequency, rectal bleeding) – excluding the endoscopic components^30^. In order to achieve a comprehensive characterization of the study group, disease activity for UC patients was furthermore assessed with the pediatric ulcerative colitis activity index (PUCAI)^31^.

The pediatric Crohn’s disease activity index (PCDAI) was used to assess the burden of disease for 19 CD patients. Similarly to the partial Mayo score in UC, there is a less invasive form of the PCDAI: the abbreviated PCDAI, which has been shown to correlate well with the complete PCDAI^32^. Accordingly, it was utilized for all subjects, in which invasive PCDAI-required diagnostics have been obtained more than four weeks prior to the echocardiographic study (n = 22).

### Conventional echocardiography

All enrolled subjects underwent a comprehensive echocardiographic study including spectral and color flow Doppler examinations according to the standard guidelines of the American Heart Association^33^. The commercially available ultrasound device iE33 by Phillips Ultrasound Inc., USA, with a S5-1 Sector Array transducer (Sector 1–5 MHz) was used. All images were digitally recorded and subsequently transferred to an offline workstation for analysis, using XCelera Version 3.1.1.422 by Phillips Ultrasound Inc., USA. Image acquisition was carried out in the apical 4-, 3- and 2-chamber views, the parasternal long axis view and in two short axis views at the mitral level and at the level of the papillary muscles. M-mode images were obtained at the level of the aortic valve and the LV for subsequent measurement of aortic root diameter, left atrial diameter, interventricular septum, LV cavity and LV posterior wall. Fractional shortening, LV mass, relative wall thickness, LV enddiastolic/endsystolic volume, EF, stroke volume and cardiac output were assessed. Utilizing pw-Doppler and pw-TDI E/A-ratio, E/E′-ratio and mitral deceleration time were detected for the assessment of LV diastolic function as described elsewhere^34^. All echocardiographic parameters were evaluated utilizing Z-scores^35^.

### Speckle tracking echocardiography

Standard cross-sectional 2D grayscale LV images were acquired for myocardial deformation (strain and strain rate) analyses. Using conventional B-Mode imaging longitudinal strain and strain rate were measured in standard apical 4-chamber (AP4), 3-chamber (AP3) and 2-chamber (AP2) views as previously described in detail^36^. Specifically, circumferential strain (CS) was measured in the standard parasternal short-axis at the mitral valve plane (SAXB) and the papillary muscle plane (SAXM). As recently suggested, five consecutive cardiac cycles synchronized to a continuous ECG were recorded with frame rate adjusted between 60 and 90 frames per second^37^. To achieve accurate deformation parameters, a special focus was set to avoid noise and minimize artifacts during the entire process of echocardiographic image acquisition. Data was anonymized, digitally stored in DICOM format and transferred to an off-line workstation for postprocessing utilizing the commercially available software QLAB Version 10. All echocardiographic examiners and interpreters were blinded to the study group status of the participants. Global and segmental strain and strain rate were assessed in seven segments per view for longitudinal strain (LS) and six segments for CS by manual tracing of the endocardial border line at end-systole. Tissue tracking quality was verified in real-time and full thickness coverage of the myocardium including the endocardial and epicardial contours was readjusted manually where necessary. Inter- and intraobserver variability was assessed by additional evaluation of resting and exercise echocardiographic images by a second independent interpreter, who was blinded to the study group status and the results of the first echocardiographic reader. The results were reproducible and inter-/intrarater variability was below 6%.

### Speckle tracking stress echocardiography

To unmask potential abnormalities in myocardial performance that might remain undiscovered at rest, we additionally exposed the study population to bicycle ergometer stress testing and performed STE. The children were asked to peddle in a supine position on a standard bicycle ergometer at 60 rounds per minute against a ramp protocol with inclining resistance. Echocardiographic images were acquired at an intermediate (approximately 0.5–1 Watt per kilogram body weight) and at the maximum level of physical exhaustion (approximately 2 Watts per kilogram body weight). A standardized pattern of consecutive images was acquired at the above-mentioned viewing planes. Peripheral blood pressure measurements were obtained at 2-minute intervals and a 12-channel ECG was continuously monitored.

### Biostatistical analyses

Demographics, clinical parameters, hemodynamic and echocardiographic data were described as mean and standard deviation. Clinical, hemodynamic and echocardiographic data of the three groups were compared utilizing the ANOVA Test. P-values < 0.05 constituted statistical significance. The data distribution was graphically displayed using Box-Whisker-Plots. Linear regression analyses and Pearson’s correlation were performed to assess the potential association of echocardiographic variables and clinical parameters. GraphPad Prism Version 6 (GraphPad Software, Inc., La Jolla, CA, USA) and Microsoft Excel Version 16.0 for PC were used for all statistical analyses.

## Results

### Patient characteristics

Baseline clinical characteristics are outlined in Table 1. Study and control groups did not differ significantly regarding age, gender distribution, body weight, height, body-mass-index, exercise routine level or Tanner stage. Disease duration was 2.6 ± 2.6 years in patients with active IBD and 3.0 ± 3.4 years in IBD patients in remission (p = 0.37). Disease activity parameters showed marked differences between patients with active disease and clinical remission both for patients with CD (PCDAI and SES-CD: 24.4 ± 18.9 and 2.7 ± 2.6 versus 10.7 ± 6.7 and 0.9 ± 0.7, respectively; p = 0.003) as well as patients with UC (Mayo endoscopic index 7.35 ± 7.5 versus 2.0 ± 2.5; p = 0.065). Furthermore, serum C-reactive protein was significantly increased in the active disease group (46.4 ± 35.6 mg/l) when compared to IBD patients in remission (21.6 ± 14.3 mg/l; p = 0.006). Hemodynamic parameters did not differ significantly between the control group and patients with active IBD. Children with IBD in remission exhibited slightly, yet statistically significant differences in heart rate (67.8 ± 9.0 beats per minute (bpm) versus 72.6 ± 9.1 bpm in healthy controls, p = 0.029) and systolic blood pressure (109 ± 10.7 mmHg versus 118.4 ± 9.9 in patients with active IBD, p = 0.025). However, these hemodynamic parameters were still within normal limits and did not reach pathologic levels.

**Table 1:** Baseline clinical characteristics and hemodynamics of the study population.

		IBD inflammed (n = 18)	IBD in remission (n = 32)	Control (n = 60)	p-value (ANOVA)
	**Age** (years)	14.58 ± 2.51	14.3 ± 2.31	14.01 ± 2.52	0.507
	**Height** (cm)	167.58 ± 16.5	161.81 ± 13.36	162.00 ± 14.38	0.735
	**Weight** (kg)	57.29 ± 18.14	54.78 ± 14.29	58.06 ± 17.88	0.749
	**Body surface** (m^2^)	1.53 ± 0.30	1.49 ± 0.29	1.57 ± 0.30	0.245
	**Body mass index** (kg/m^2^)	20.02 ± 4.59	20.63 ± 3.93	21.61 ± 4.35	0.227
	**Exercise routine** (1 = in school; 2 = <3 times/week; 3 = ≥3 times/week)	1.70 ± 0.72	1.90 ± 0.87	1.78 ± 0.73	0.83
	**Tanner stage**	3.47 ± 1.45	3.18 ± 1.09	3.33 ± 1.13	0.765
	**Duration of disease (**years)	2.56 ± 2.56	3.00 ± 3.44	/	0.37
	**PCDAI (CD)**	24.37 ± 18.91	10.70 ± 6.70	/	0.157
	**SES-CDIleum (CD)**	2.71 ± 2.56	0.88 ± 0.66	/	0.003
	**MAYO endoscopic index (UC)**	7.25 ± 7.5	2.00 ± 2.52	/	0.065
	**CU disease extent** (None/Proctitis/left sided colitis/extensive colitis)	16/2/3/4	/	/	/
	**PUCAI Index**	40.00 ± 17.14	2.89 ± 4.87	/	<0.001
	**Extraintestinal manifestations**	4	10	/	/
	**Serum C-reactive protein level** (mg/l)	46.4 ± 35.6	21.6 ± 14.3	/	0.006
At rest	**Heart rate** (bpm)	74.55 ± 10.99	67.84 ± 9.01	72.61 ± 9.10	0.029
	**BP systolic** (mmHg)	118.44 ± 9.85	109 ± 10.70	112.61 ± 12.22	0.025
	**BP diastolic** (mmHg)	68.22 ± 7.55	64.21 ± 7.59	65.1 ± 8.22	0.23
Stress testing	**Heart rate** (bpm)	153.61 ± 13.17	142.71 ± 16.18	151.07 ± 15.16	0.023
	**BP systolic** (mmHg)	135.06 ± 5.62	133.31 ± 9.24	136.48 ± 18.88	0.625
	**BP diastolic** (mmHg)	78.12 ± 7.99	75.46 ± 8.01	74.98 ± 10.95	0.531
	**Level of resistance** (W/kg body weight)	1.53 ± 0.49	1.48 ± 0.59	1.58 ± 0.47	0.686

#SES-CD = Simple Endoscopic Score

Most patients were on oral 5-ASA (n = 10 in active IBD; n = 16 for IBD in remission), corticosteroids (n = 4 in active IBD; n = 13 for IBD in remission) and azathioprine (n = 4 in active IBD; n = 8 for IBD in remission). Other IBD medications used included TNF-alpha inhibitors, 6-mercaptopurine, cyclosporine and probiotics (Table S1). Conventional abdominal ultrasound revealed several differences between the two study sub-groups (Table S2). Children with active IBD had significantly increased terminal ileum wall thickness (3.6 ± 1.58 mm) when compared to children with IBD in remission (2.22 ± 2.01) (p = 0.001). Furthermore, superior mesenteric artery blood flow (116.65 ± 42.53 cm/s versus 108.63 ± 39.14 cm/s) and superior mesenteric artery diameter (6.29 ± 0.64 mm versus 5.60 ± 0.76) was higher in patients with active disease than in those patients with IBD in remission.

### Conventional echocardiographic parameters

Most conventional echocardiographic parameters including LV ejection fraction and estimated LV mass were similar in children with IBD and in healthy controls (Table 2). Patients with IBD in remission had a slightly lower left atrium to aortic root ratio (0.93 ± 0.07) and an increased interventricular septal end-diastolic diameter (0.94 ± 0.19 cm) when compared to those with active IBD and healthy volunteers (p = 0.001). However, these parameters were within normal limits as evaluated by Z-scores. Interestingly, both children with active IBD (−8.57 ± 1.14 cm/s) and those with IBD in remission (−8.66 ± 1.30 cm/s) showed a statistically significant increase in E/E′ ratio in comparison to healthy controls (−7.43 ± 2.87 cm/s, p = 0.031). Carotid intima-media-thickness was similar in IBD patients and control subjects (p = 0.572).

**Table 2:** Conventional echocardiographic parameters derived from two-dimensional and Doppler imaging.

	IBD inflammed (n = 18)	IBD in remission (n = 32)	Control (n = 60)	p-value (ANOVA)
LA/AoR	1.01 ± 0.10	0.93 ± 0.07	1.03 ± 0.12	0.001
Fractional shortening (%)	32.91 ± 3.48	34.53 ± 3.92	34.51 ± 3.65	0.283
Interventricular septal end-diastolic diameter (cm)	0.87 ± 0.16	0.94 ± 0.19	0.85 ± 0.15	0.046
LV end-diastolic diameter (cm)	4.33 ± 0.37	4.47 ± 0.41	4.36 ± 0.61	0.559
LV posterior wall diameter. diastolic (cm)	0.85 ± 0.12	0.97 ± 0.58	0.88 ± 0.21	0.422
LV mass (g)	121.43 ± 35.57	130.06 ± 40.09	129.03 ± 49.69	0.799
Relative wall thickness	0.19 ± 0.02	0.19 ± 0.03	0.23 ± 0.18	0.431
End-diastolic volume of the left ventricle (ml)	119.03 ± 37.84	117.83 ± 42.02	107.77 ± 36.84	0.385
Ejection fraction (%)	61.24 ± 3.53	60.26 ± 3.18	60.66 ± 5.14	0.768
Stroke volume (ml)	73.54 ± 21.18	71.84 ± 24.27	65.95 ± 24.42	0.383
E-Wave/A-Wave	1.8 ± 0.30	1.85 ± 0.41	1.85 ± 0.33	0.830
Mitral deceleration time (s)	0.15 ± 0.02	0.16 ± 0.02	0.17 ± 0.04	0.056
E/E′ (cm/s)	−8.57 ± 1.14	−8.66 ± 1.30	−7.43 ± 2.87	0.031
Carotid intima-media-thickness (mm)	0.38 ± 0.07	0.40 ± 0.09	0.41 ± 0.09	0.572

### Speckle tracking stress echocardiography

**Figure 1:**
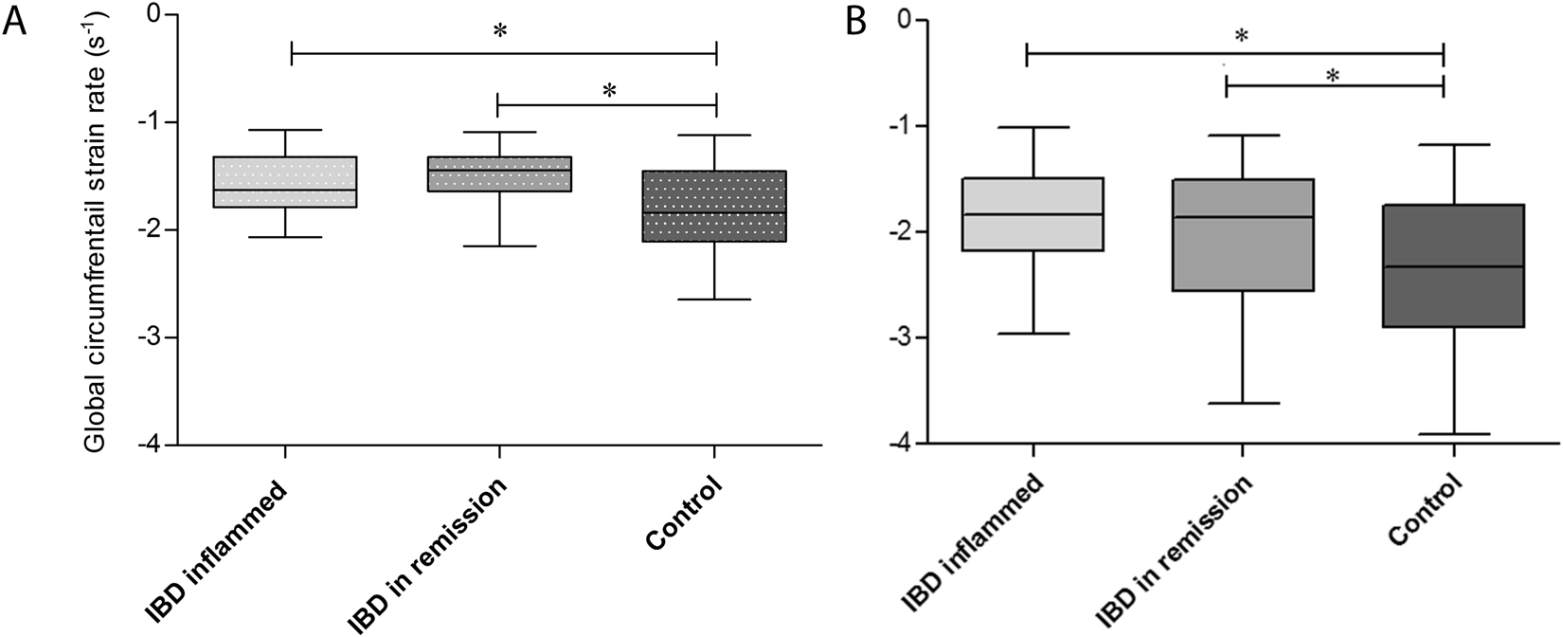
Global LV peak circumferential strain rate at rest (**left**) and during exercise testing (**right**)

**Figure 2:**
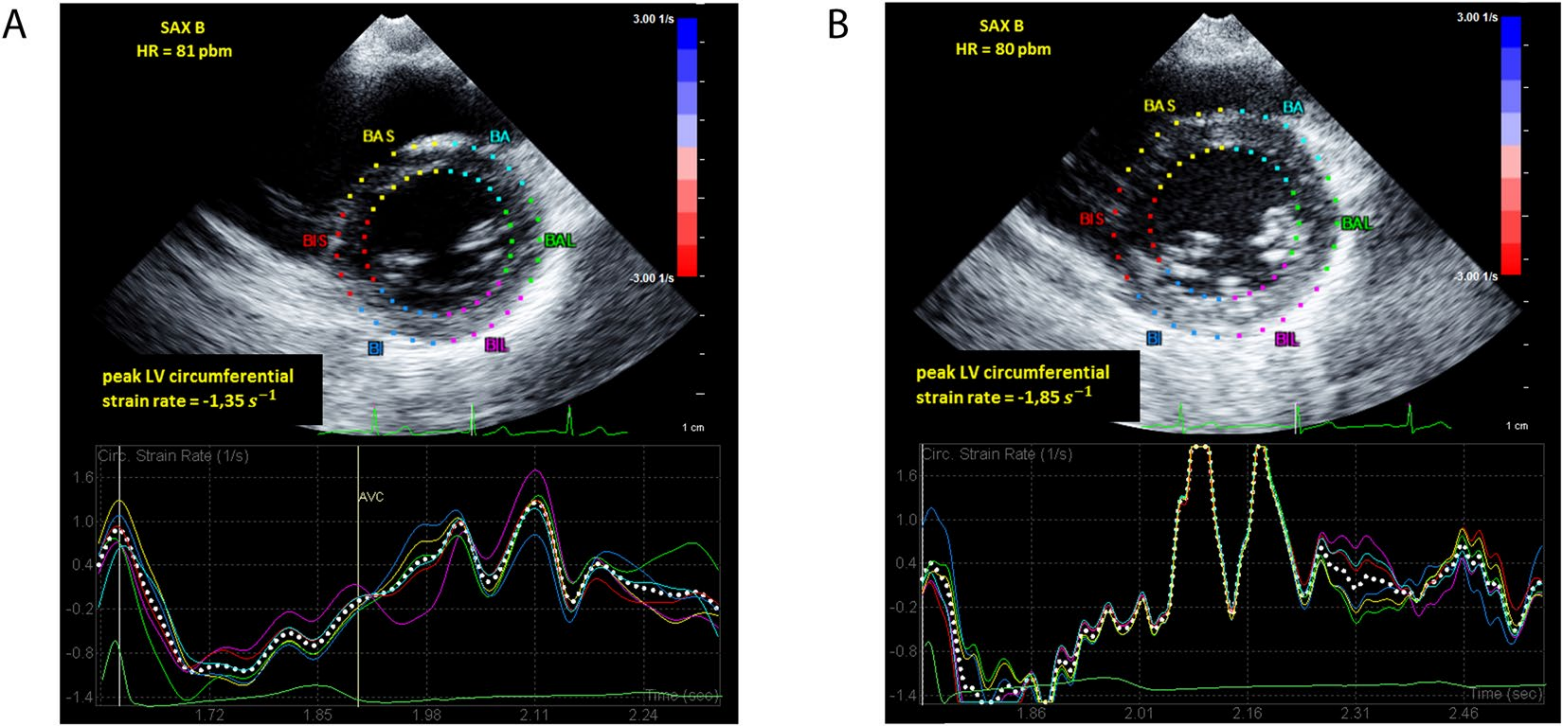
Echocardiographic short axis view derived circumferential strain rate at rest in a patient with active CD (**left**) and a healthy control (**right**). *Note the significantly decreased peak LV circumferential strain rate in the child with IBD*.

Overall, children with active IBD and children with IBD in remission exhibited decreased levels of peak LV circumferential and longitudinal strain rate (Table 3, Fig. 1) and strain (Table 4) when compared to healthy controls both at rest and during exercise testing. In detail, LV peak global circumferential strain rate was markedly depressed in patients with active IBD (−1.55 ± 0.26 s^−1^) and those with IBD in remission (−1.49 ± 0.26 s^−1^) in comparison to healthy volunteers (−1.8 ± 0.4) at rest (p = 0.001). A representative echocardiographic example is demonstrated in Fig. 2. Accordingly, this difference was also detected during stress testing with significantly lower global circumferential strain rate in active IBD (−1.84 ± 0.5 s^−1^) and IBD in remission (−1.95 ± 0.57 s^−1^, versus −2.36 ± 0.69 s^−1^ in the control group, p = 0.022). Similarly, longitudinal strain rate was decreased in patients with IBD throughout all analyzed echocardiographic view planes both at rest and during stress testing without reaching statistical significance (Table 3).

**Table 3:** Speckle tracking derived peak systolic LV strain rate at rest and during stress testing.

		IBD inflamed (n = 18)	IBD in remission (n = 32)	Control (n = 60)	p-value (ANOVA)
At rest	**Global circumferential strain rate** (s^−1^)	−1.55 ± 0.26	−1.49 ± 0.26	−1.80 ± 0.4	0.001
	**Circumferential strain rate (SAXM)** (s^−1^)	−1.58 ± 0.36	−1.51 ± 0.27	−1.78 ± 0.47	0.011
	**Circumferential strain rate (SAXB)** (s^−1^)	−1.51 ± 0.3	−1.48 ± 0.28	−1.80 ± 0.48	0.001
	**Global longitudinal strain rate** (s^−1^)	−1.31 ± 0.28	−1.33 ± 0.36	−1.41 ± 0.36	0.458
	**Longitudinal strain rate (AP4)** (s^−1^)	−1.30 ± 0.27	−1.31 ± 0.4	−1.40 ± 0.39	0.464
	**Longitudinal strain rate (AP2)** (s^−1^)	−1.24 ± 0.29	−1.30 ± 0.42	−1.38 ± 0.37	0.364
	**Longitudinal strain rate (AP3)** (s^−1^)	−1.38 ± 0.42	−1.36 ± 0.37	−1.42 ± 0.42	0.797
Stress testing	**Global circumferential strain rate** (s^−1^)	−1.84 ± 0.5	−1.95 ± 0.57	−2.36 ± 0.69	0.022
	**Circumferential strain rate (SAXM)** (s^−1^)	−1.82 ± 0.36	−1.93 ± 0.57	−2.18 ± 0.74	0.127
	**Circumferential strain rate (SAXB)** (s^−1^)	−2.05 ± 0.74	−1.94 ± 0.67	−2.27 ± 0.7	0.283
	**Global longitudinal strain rate** (s^−1^)	−1.81 ± 0.4	−1.72 ± 0.43	−1.95 ± 0.49	0.123
	**Longitudinal strain rate (AP4)** (s^−1^)	−1.78 ± 0.4	−1.71 ± 0.53	−1.92 ± 0.62	0.326
	**Longitudinal strain rate (AP2)** (s^−1^)	−1.82 ± 0.46	−1.72 ± 0.49	−2.05 ± 0.55	0.05
	**Longitudinal strain rate (AP3)** (s^−1^)	−1.84 ± 0.47	−1.71 ± 0.47	−2.03 ± 0.57	0.073

**Table 4:** Speckle tracking derived peak systolic LV strain at rest and during stress testing.

		IBD inflamed (n = 18)	IBD in remission(n = 32)	Control (n = 60)	p-value(ANOVA)
At rest	**Global circumferential strain** (%)	−18.58 ± 2.43	−20.21 ± 2.87	−21.50 ± 4.25	0.133
	**Circumferential strain (SAXM)** (%)	−18.48 ± 3.98	−21.20 ± 2.87	−22.21 ± 4.45	0.123
	**Circumferential strain (SAXB)** (%)	−18.68 ± 3.06	−19.21 ± 3.35	−20.47 ± 4.11	0.159
	**Global longitudinal strain** (%)	−19.96 ± 3.16	−20.00 ± 2.22	−19.59 ± 3.46	0.807
	**Longitudinal strain (AP4)** (%)	−20.48 ± 3.55	−20.42 ± 2.66	−19.62 ± 2.4	0.305
	**Longitudinal strain (AP2)** (%)	−19.85 ± 3.59	−19.78 ± 2.62	−19.37 ± 6.47	0.916
	**Longitudinal strain (AP3)** (%)	−19.67 ± 3.56	−20.04 ± 2.61	−19.98 ± 3.3	0.924
Stress testing	**Global circumferential strain** (%)	−18.67 ± 3.97	−17.83 ± 3.57	−19.32 ± 3.62	0.285
	**Circumferential strain (SAXM)** (%)	−19.34 ± 4.25	−18.65 ± 4.22	−19.77 ± 3.74	0.568
	**Circumferential strain (SAXB)** (%)	−17.62 ± 4.15	−17.49 ± 3.55	−18.40 ± 3.29	0.679
	**Global longitudinal strain** (%)	−20.52 ± 3.63	−19.61 ± 2.88	−19.66 ± 2.59	0.566
	**Longitudinal strain (AP4)** (%)	−20.43 ± 4.14	−20.30 ± 3.21	−20.27 ± 3.34	0.988
	**Longitudinal strain (AP2)** (%)	−20.64 ± 3.76	−19.61 ± 3.31	−19.38 ± 2.79	0.445
	**Longitudinal strain (AP3)** (%)	−20.48 ± 4.29	−18.96 ± 4.45	−19.71 ± 2.97	0.456

Results from LV strain analyses showed an analogical tendency of continuously depressed circumferential myocardial performance in IBD versus healthy controls both at rest and during bicycle ergometry testing. However, these differences were not statistically significant. Peak LV longitudinal strain was unchanged in children with IBD and healthy controls in the resting (p = 0.807) and exercise state (p = 0.566). LV myocardial performance parameters did not differ significantly between children with CD and UC (*data not shown*).

**Figure 3:**
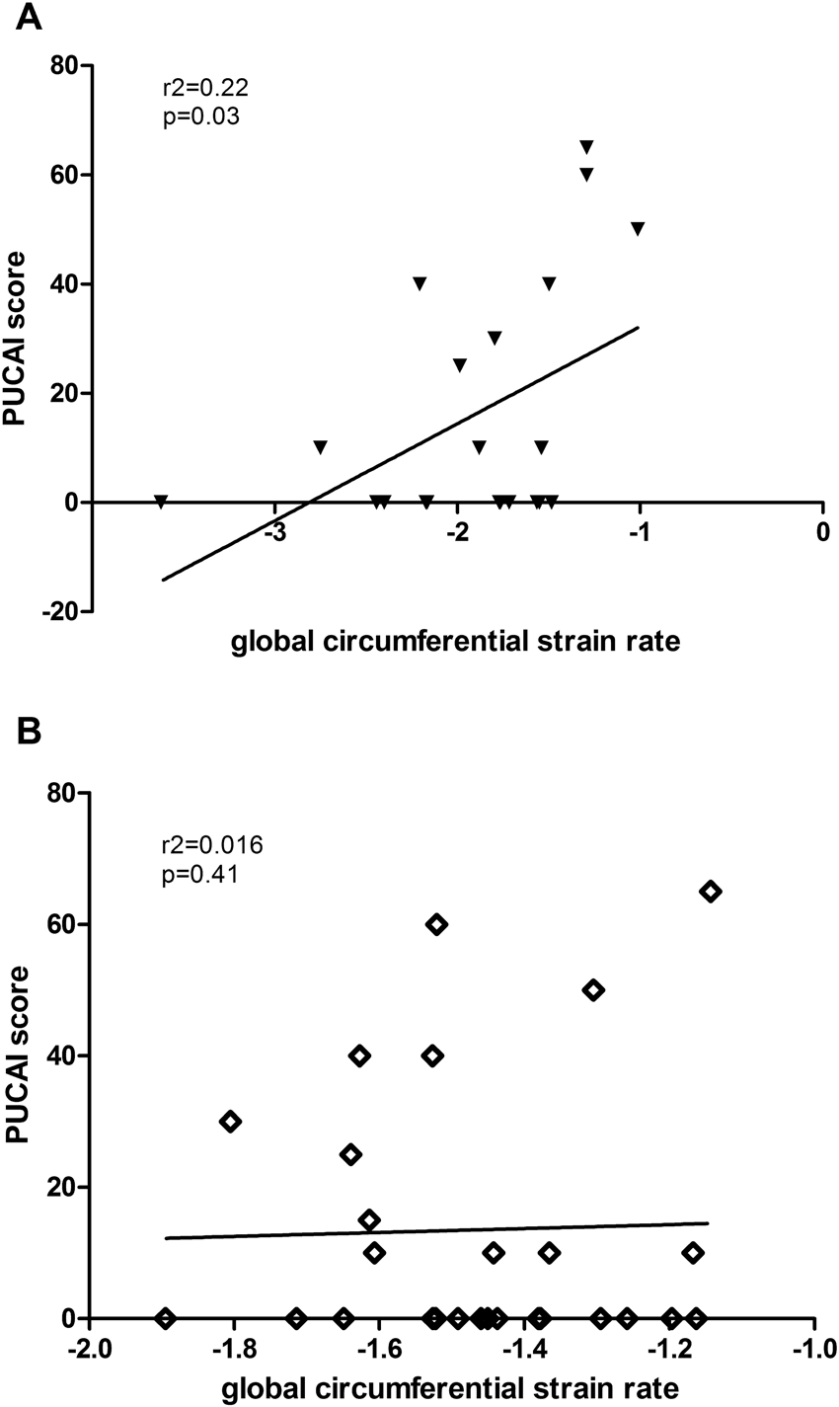
Correlation analysis between PUCAI and LV peak circumferential strain rate during exercise (**A**) and at rest in pediatric UC patients (**B**).

Correlation and linear regression analyses did not show any significant associations between echocardiographic and clinical or hemodynamic parameters except for global circumferential strain rate under stress and CrP and PUCAI in patients with UC (Table 5). For the latter, we observed a weak direct linear correlation (Fig. 3, r2 = 0.22, p = 0.03). Global circumferential strain rate at rest, however, did not correlate with PUCAI (Fig. 3, r2 = 0.016, p = 0.41). Interestingly no such correlation was observed in patients with CD.

**Table 5:** Correlation analyses of disease activity scores, basic laboratory and abdominal ultrasound findings with global LV circumferential strain rate at rest and during exercise in pediatric IBD patients using Pearson’s correlation analysis.

	Global LV circumferential strain rate at rest		Global LV circumferential strain rate during exercise	
	r	p-value	r	p-value
PCDAI (CD)	0.13	0.61	−0.05	0.87
SES-CDIleum (CD)	0.18	0.49	0.09	0.77
MAYO endoscopic index (UC)	−0.19	0.38	−0.001	0.99
PUCAI Index	0.029	0.89	0.47	0.03
Duration of disease	−0.08	0.57	−0.04	0.80
Serum C-reactive protein level	−0.15	0.31	−0.38	0.021
Erythrocyte sedimentation rate	−0.13	0.45	0.01	0.96
Fecal calprotectin	−0.13	0.41	0.06	0.72
Terminal ileum wall thickness	−0.02	0.93	−0.02	0.95
Superior mesenteric artery blood flow	0.15	0.38	−0.01	0.95
Superior mesenteric artery diameter	−0.01	0.95	−0.24	0.22

## Discussion

To assess the effect of IBD on LV myocardial performance in pediatric patients at an early disease phase, we performed speckle tracking echocardiography (STE) in combination with ergometer stress testing in asymptomatic normotensive pediatric patients with IBD and healthy controls. To determine the significance of inflammatory disease activity, a priori, IBD patients were stratified into subgroups of children with active disease and IBD in remission. The reason for this subdivision was the underlying concept of inflammation as a potential impairing factor for myocardial contractility in these patients. Cardiac impairment has been demonstrated before in other autoimmune inflammatory diseases such as psoriasis^38, 39^, rheumatoid arthritis^40^ or systemic lupus erythematodes^41^.

In the present study children with IBD showed significantly impaired LV myocardial performance parameters when compared to healthy controls (Table 3). Specifically, circumferential strain rate was depressed at rest (p = 0.001) and more pronounced during exercise testing (p = 0.022). This is in agreement with a recent STE study in adults, demonstrating depressed LV strain and strain rate^24^. In detail, the authors report of altered longitudinal deformation, while circumferential strain rate was not assessed. In our study, longitudinal strain rate was also impaired in IBD patients, without reaching a degree of statistical significance (p = 0.123). Our findings are further in line with a conventional two-dimensional echocardiographic study from 2016, in which subclinical cardiac involvement was demonstrated in adult patients with CD and UC^25^. Interestingly, while the study reports IBD patients to feature significantly lower left ventricular ejection fraction and altered enlarged LV morphologic parameters, there was no difference between CD and UC patients, which is also in accordance with findings from the present study (*data not shown*). As disease duration was short (on average ≤ 3 years) in our study cohort, it is not surprising that only subtle changes of LV performance were detectable and not all dimensions of myocardial deformation exhibited the same degree of changed cardiac dynamics. Importantly and without exception, LV peak circumferential strain rate was depressed in IBD patients both at rest and during exercise testing in all analyzed echocardiographic view planes representing the entirety of the LV (Table 3). Potential underlying pathomechanisms of IBD-mediated cardiac disease include microvascular endothelial dysfunction due to disturbed nitric oxide (NO)-driven vasoregulation^42^, a shift from endothelial production of NO and endothelial-derived hyperpolarizing factor to nonendothelial vascular tissue^43^ and disturbed collagen metabolism^44^. However, these mechanisms are currently poorly understood and warrant further experimental investigations.

The phenomenon of significantly altered LV peak strain rate and merely unchanged cardiac strain can be explained by two co-occurring mechanisms. Strain describes the percent change in systolic (and diastolic) myocardial length in relation to its end-diastolic state (end-systolic state, respectively). Strain rate is the temporal integration of strain, measured in s^−1^. Because strain has been shown to be confounded by cardiac loading conditions (e.g. Frank-Starling mechanism), strain rate is considered the more robust index for the non-invasive quantification of LV contractility^45, 46^. Therefore, firstly, strain values may have likely been more distorted and thus resulted in less pronounced differences between the analyzed groups. Secondly, as atrial electromechanical conduction has also been shown to be prolonged in IBD, and exposure to chronic inflammation may result in structural and electrophysiological changes in the atrial tissue that causes slow conduction^47^. A similar pathomechanism can likely take place in the ventricles as well. We hypothesize, that IBD-mediated alterations of cardiac mechanics may cause a delay in myocardial fiber deformation rather than overtly impaired total LV contractility (as reflected by preserved LVEF and fractional shortening). Delayed LV peak myocardial contractility can be measured using strain rate, but not with strain.

Curiously, one patient was initially enrolled as an asymptomatic healthy control subject. 3 months later, she became symptomatic with abdominal pain and recurrent diarrhea. Upon diagnostic work-up she received the diagnosis of CD and was included into the study group with a disease duration of 5.5 months (Figure S1). Interestingly, this example demonstrated a substantial decrease in LV circumferential and longitudinal strain rate both at rest and during exercise after onset of CD in this patient. While this phenomenon could only be observed in a single patient and is therefore statistically not representative, it does reflect the above described impact of IBD on LV performance.

Interestingly, both pediatric patients with active IBD and those with IBD in clinical remission exhibited signs of depressed myocardial performance. While several viewing planes revealed lower LV deformation in inflamed IBD, overall, there were no marked differences between the two subgroups. At first, this might seem somewhat counterintuitive given the concept that inflammatory activity ought to be the most relevant causative factor for cardiac alterations in these patients – e.g. recent studies have demonstrated IBD activation to feature significantly depressed coronary flow reserve^48^. Furthermore, CD was associated with impairment in LV global longitudinal myocardial function and Crohn’s Disease Activity Index was correlated with LV global longitudinal strain in in adult patients^26^. Strain rate was not assessed in that study. In the present study, strain and strain rate in patients with active inflammation and those with IBD in remission did not differ widely with the exception of a weak correlation between PUCAI and global circumferential strain rate under stress in UC patients; most probably due to the discrete nature of the cardiac alterations in these patients. Hence, myocardial impairment in this patient population may be too subtle to result in overt differences between inflamed IBD and patients in clinical remission. The key difference between this study and other studies investigating LV performance in IBD patients is firstly, that our study cohort consists of children and secondly the short disease duration of less than three years on average. Subsequently, in this short time span myocardial performance in IBD patients in remission may not have recuperated (yet) to a superior level when compared to patients with active IBD. Therefore, marked differences that have been observed in adult patients with longer disease duration may not be detectable in pediatric patients in the early disease phase. However, even though the observed correlation between disease activity and global circumferential strain rate in UC patients is discrete and should not be over-interpreted, it might constitute an early sign of this association. Follow-up studies must be performed to assess the dynamic evolvement of myocardial performance in relation to IBD activity.

The E/E′ ratio was significantly increased in IBD patients (p = 0.031) as compared to healthy controls (Table 2), representing a subtle sign of beginning diastolic malfunction. This is in accordance with results from two studies of 2015 and 2016, in which the authors have detected impaired coronary microvascular and LV diastolic function in patients with IBD^49, 50^. Similarly to the above outlined rather discrete differences in LV systolic performance indices between IBD patients and healthy volunteers, diastolic dysfunction also only manifests in a subtle manner. Therefore, E/A ratio was most probably not yet significantly changed in IBD patients as disease influence was not (yet) long-acting in this study population. Nevertheless, it is still both surprising and important that impaired myocardial relaxation can already be measured in patients with IBD duration of less than three years. This is further in agreement with another study from 2016 reporting of degenerated LA volume and mechanical functions and increased atrial electromechanical delay in adult patients with UC – all of which was related to duration of disease^51^.

As expected, conventional echocardiography yielded only minor differences between the analyzed study groups. We observed a slightly increased statistically significant, yet clinically irrelevant interventricular septal end-diastolic diameter in IBD patients (Table 2). The use of corticosteroids in these patients is a possible explanation for this finding, as septal hypertrophy has been observed before in steroid use^52^. Nevertheless, the biological relevance of this finding is unlikely to be high as there were no statistical differences of posterior wall diameter, end-systolic septal diameter or overall estimated LV mass between the study groups and all conventional echocardiographic parameters were within normal limits as evaluated by Z-scores.

UC in adults has been associated with both increased arterial stiffness and carotid intima-media thickness^19^. Moreover, in a pediatric study, IBD patients showed signs of premature endothelial dysfunction, increased carotid intima-media thickness (IMT) and decreased flow-mediated dilation of brachial arteries^17^. In contrast, IMT was not significantly different between the study groups. This is most probably due to shorter disease duration in our study cohort and the use of a standard b-mode ultrasound device which may not be sensitive enough to assess subtle changes in carotid artery IMT (vs. high-resolution ultrasound device utilized by Aloi *et al*.).

## Conclusion

Pediatric patients with CD and UC show subclinical signs of impaired LV systolic and diastolic myocardial performance early in the course of the disease. LV function is altered both in patients with active inflammation and in IBD in remission. Long-term studies are needed to verify these subtle findings and to build correlations to clinical outcome parameters. This underlines the importance of cardiovascular prevention in the day-to-day clinical care of patients with IBD.

## Electronic supplementary material

Tables S1–2 and Figure S1
