# Supplementary material for: Speckle Tracking Stress Echocardiography Uncovers Early Subclinical Cardiac Involvement in Pediatric Patients with Inflammatory Bowel Diseases
Source: Sci Rep. 2017 Jun 7;7:2966. doi: 10.1038/s41598-017-03255-1 (PMC5462781; doi:10.1038/s41598-017-03255-1)
Supplement: Supplementary file 1 — Tables S1–2 and Figure S1 [file 41598_2017_3255_MOESM1_ESM.doc]

**Manuscript Supplemental Material**

**Speckle Tracking Stress Echocardiography Uncovers Early Subclinical Cardiac Involvement in Pediatric Patients with Inflammatory Bowel Diseases**

Kai O. Hensel^1,2,*^, Francisca E. Abellan Schneyder^2^, Lucia Wilke^2^, Andreas Heusch^2^, Stefan Wirth^1^ and Andreas C. Jenke^3^

^1^ *HELIOS University Medical Center Wuppertal, Children’s Hospital, Department of Pediatric Gastroenterology, Center for Clinical & Translational Research (CCTR), Faculty of Health, Center for Biomedical Education & Research (ZBAF), Witten/Herdecke University, Faculty of Health, Germany*

^2^ *HELIOS University Medical Center Wuppertal, Children’s Hospital, Department of Pediatric Cardiology, Center for Clinical & Translational Research (CCTR), Faculty of Health, Center for Biomedical Education & Research (ZBAF), Witten/Herdecke University, Faculty of Health, Germany*

^3^ *EKO Children's Hospital, Department of Pediatric Gastroenterology, Oberhausen, Witten/Herdecke University, Faculty of Health, Germany*

Short title: Speckle tracking stress echo in pediatric IBD

**Table S1.** Medications of IBD patients.

|  | **IBD  inflammed**  (n=18) | **IBD  in remission**  (n=32) |
| --- | --- | --- |
| **TNF-alpha inhibitors** (n) | 1 | 0 |
| **Oral 5-ASA** (n) | 10 | 16 |
| **Azathioprin** (n) | 4 | 8 |
| **Corticosteroids** (n) | 4 | 13 |
| **Probiotics** | 1 | 1 |
| **6-Mercaptopurin** | 1 | 1 |
| **Ciclosporin** | 1 | 0 |

**Table S2.** Abdominal ultrasound in IBD patients

|  | **IBD  inflammed**  (n=18) | **IBD  in remission**  (n=32) | **p-value** |
| --- | --- | --- | --- |
| **Terminal ileum wall thickness** (mm) | 3.6±1.58 | 2.22±2.01 | 0.001 |
| **Superior mesenteric artery blood flow** (cm/s) | 116.65±42.53 | 108.63±39.14 | 0.144 |
| **Superior mesenteric artery diameter** (mm) | 6.29±0.64 | 5.60±0.76 | 0.511 |


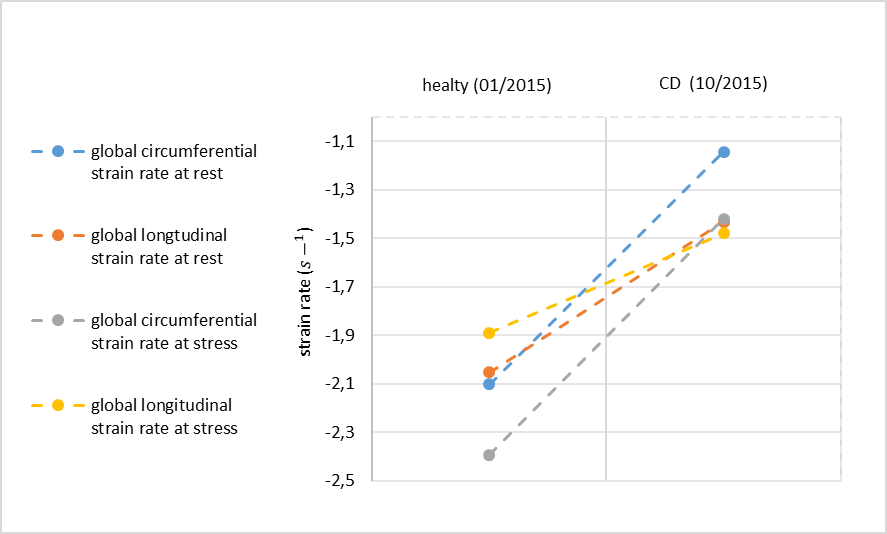


**Figure S1.** Global LV longitudinal and circumferential peak strain rate at rest **(left)** and during exercise **(right)** in a 14-year-old girl who was first enrolled as a healthy control and 9.5 months later again as an IBD patient in the study group after she happened to be diagnosed with CD.
